# Management of Traumatic Injury to the Left Foot in a 48-Year-Old Man: A Case Report

**DOI:** 10.7759/cureus.85695

**Published:** 2025-06-10

**Authors:** Joshua A Jogie, David King, Donna Rampersad, Danielle Karim, Tounesha La Rosa

**Affiliations:** 1 Occupational Health Unit, St. James Medical Complex, Port of Spain, TTO; 2 Internal Medicine, St. James Medical Complex, Port of Spain, TTO; 3 Accident and Emergency, St. James Medical Complex, Port of Spain, TTO

**Keywords:** emergency care, occupational health, physical medicine and rehabilitation, traumatic foot injury, wound management

## Abstract

Approaches to foot injury care depend on a range of clinical steps that promote proper healing and function. We present a case of a 48-year-old plumber who injured his left foot when a metal object fell on it at home. Initial management involved irrigation, suturing, antibiotics, analgesia, and a plain foot X-ray, which was normal. Follow-up at the Occupational Health Unit (OHU) addressed persistent swelling, pain, and numbness. Further evaluation ruled out a fracture, but the patient required extended sick leave and restrictions on weight-bearing tasks. He received nonsteroidal anti-inflammatory drugs (NSAIDs), topical agents, vitamin supplements, and physiotherapy. Laboratory tests revealed elevated cholesterol, prompting statin therapy. Over four weeks, he progressed to resuming duties with restrictions. After seven weeks, continued monitoring by a multidisciplinary team supported his return to full duties without restrictions. This case illustrates the value of thorough wound care, tailored pain control, and coordinated care among multiple services. The prompt detection of comorbidities such as hyperlipidemia enabled early intervention. Ultimately, a stepwise combination of medical treatment, rehabilitation, and workplace adaptations restored the patient’s full function and ability to perform his usual work.

## Introduction

Foot injuries resulting from blunt trauma or penetrating wounds are common occurrences in both occupational and domestic settings. These injuries often involve direct impact with heavy or sharp objects and frequently affect the soft tissues, bones, and nerves of the foot, leading to varying degrees of morbidity and prolonged functional impairment [1,2]. Prompt and appropriate initial management is critical to prevent complications such as infection, chronic pain, nerve injury, and prolonged absence from occupational activities [3]. Even small lacerations or contusions can progress to chronic pain, infection, or nerve damage when treatment is delayed or inadequate, leading to long-term disability and extended recovery [4]. The aim of this case report is to detail the seven-week multidisciplinary management and functional recovery of a foot laceration in an occupational setting. This sets it apart from existing studies that focus on single-discipline management.

Occupational foot injuries have a significant impact on workers, particularly those whose jobs involve physical labor, prolonged standing, climbing, and exposure to environmental hazards [5]. Plumbers, construction workers, and manual laborers are especially susceptible to foot injuries due to their frequent exposure to heavy equipment, sharp tools, and strenuous physical activities. These injuries significantly affect their capacity to perform daily occupational tasks, resulting in economic consequences due to lost productivity and extended medical leave [1,5]. Understanding the mechanisms, initial evaluation strategies, and management of such injuries is crucial for occupational health units (OHUs), emergency departments (EDs), and rehabilitation specialists.

Acute foot injuries frequently present as lacerations, contusions, fractures, or a combination of these. The initial evaluation typically involves clinical examination and imaging, commonly with X-rays, to rule out fractures or foreign bodies. Even when initial imaging is negative, subtle injuries such as hairline fractures, ligamentous injuries, tendon involvement, or soft tissue damage may become apparent in subsequent evaluations. Thus, clinicians should maintain a high degree of suspicion and conduct follow-up assessments if symptoms persist beyond the typical acute healing phase [6].

Pain and swelling are common presenting symptoms of foot trauma and are initially managed with rest, ice, compression, elevation, and early diagnosis (RICED). Early diagnosis denotes prompt diagnostic assessment. This includes targeted imaging and specialist referral when indicated. This defines injury extent and guides definitive care. NSAIDs and analgesics remain standard treatments to reduce inflammation and control pain [7]. However, persistent pain and swelling beyond the expected period of acute inflammation can indicate underlying complications such as chronic soft tissue injury, delayed healing, or nerve damage. Such presentations warrant further diagnostic investigations and a multidisciplinary management strategy, including orthopedics, physiotherapy, and physical medicine and rehabilitation (PMR) [8].

Nerve injuries of the foot, though less frequently identified immediately following trauma, can significantly impact functional outcomes and patient quality of life. Peripheral nerve injuries in the foot commonly present with pain, numbness, tingling sensations, or motor dysfunction [9]. Damage to sensory nerves complicates recovery and the return to occupational activities. Early identification and management, including physiotherapy, range-of-motion and strengthening exercises, as well as pharmacological interventions, such as gabapentinoids or tricyclic antidepressants for neuropathic pain, are essential in mitigating long-term functional impairment [9].

Foot wounds also pose a high risk for infection, particularly in occupational environments involving contact with contaminated water or surfaces. Infection risks increase significantly in lacerations that are not promptly or adequately cleaned, repaired, and treated with prophylactic antibiotics, especially when patients lack updated tetanus immunization [3]. Consequently, strict adherence to wound management protocols, including irrigation, suturing, antibiotic prophylaxis, and tetanus immunization, is crucial in preventing secondary infections and complications [3]. Tetanus incidence in the United States from 2001 to 2008 averaged 0.10 cases per 1,000,000 population per year, with a case‐fatality rate of 13.2% in untreated individuals [3].

Chronic conditions such as varicose veins, peripheral vascular diseases, or diabetes mellitus further complicate healing in foot injuries. Varicose veins, frequently observed among individuals engaged in prolonged standing or physically demanding occupations, can exacerbate foot swelling, discomfort, and delayed wound healing [10]. Comprehensive assessment of pre-existing medical conditions and their impact on injury recovery is vital in occupational health management [10].

Additionally, nutritional deficiencies, particularly vitamins B1, B6, and B12, have been associated with delayed healing and increased neuropathic pain following traumatic injuries. Supplementation of these vitamins is often considered as adjunctive therapy in managing persistent neurological symptoms and promoting nerve regeneration post-injury [9].

Routine laboratory tests, including complete blood count, renal and liver function tests, lipid profiles, and blood glucose monitoring, are essential components of comprehensive care for patients with prolonged recovery from occupational injuries [6]. These investigations help in identifying underlying conditions or metabolic abnormalities that could impede the healing process or increase susceptibility to complications [6].

Foot injuries are prevalent occupational hazards with significant implications for affected individuals and their work capacities. Early recognition, adequate initial management, timely follow-up, and comprehensive rehabilitation strategies are critical components in preventing chronic complications and facilitating successful return to occupational activities. This case report discusses the clinical course and rehabilitation outcomes of a 48-year-old male plumber who sustained a foot injury from a heavy metal object at home. Although the trauma occurred outside the workplace, the resulting functional limitations directly compromised his ability to perform job-related tasks, making the case highly relevant to occupational health practice. It is reported to demonstrate how early, coordinated occupational health, emergency, and rehabilitation interventions can shorten recovery, prevent chronic disability, and inform best practices for similar foot injuries. This highlights critical aspects of occupational health care and multidisciplinary management in preventing chronic complications and ensuring successful functional recovery.

## Case presentation

A 48‐year‐old male plumber presented to the OHU soon after sustaining a lacerating injury, 10 cm in length, to his left foot at home. While working on a do-it-yourself (DIY) plumbing project at home, he lifted a heavy cylindrical steel pipe when a crescent-shaped adjustable wrench fell onto his foot. He performed initial first aid by dressing the wound himself. However, the dressing remained soaked with blood until, about one hour after the injury, he presented to the OHU for further care. He also reported that he had not received a tetanus booster for over 10 years. This prompted immediate referral to the Accident and Emergency Department (AED). At the AED, which the patient attended within two hours of the injury, the wound was thoroughly irrigated with 1 liter of normal saline (0.9% sodium chloride solution) to remove debris and reduce the risk of infection. Following irrigation, the wound was repaired using simple interrupted sutures. Eight sutures were placed to approximate the wound edges using 5-0 nylon sutures. The choice of suture and the number of sutures provided adequate tension relief and proper alignment of the tissue edges. The wound’s contaminated nature and the patient’s overdue tetanus immunization prompted the administration of tetanus prophylaxis. This included a tetanus toxoid vaccine according to the standard protocol.

**Figure 1:**
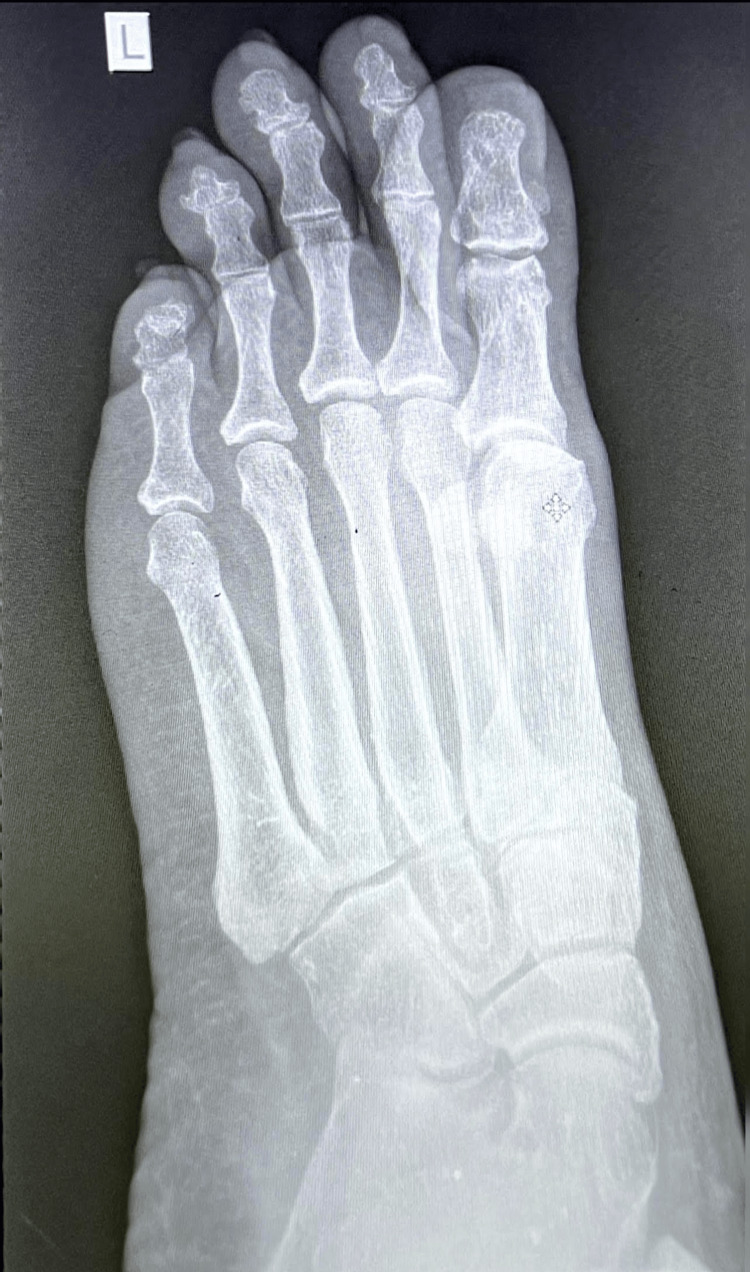
Initial AP plain X-ray of the left foot demonstrating no evidence of fracture. AP: anteroposterior.

In addition, the patient was started on prophylactic antibiotic therapy with amoxicillin-clavulanate 875/125 mg twice daily for a period of seven days. For pain control, the patient received ibuprofen 400 mg to be taken every 8 hours as needed for up to seven days. A dressing was then applied to the wound to maintain a clean environment and protect the area. An initial plain X-ray of the left foot was performed at this early stage which did not reveal any fracture or foreign body (Figure 1). An orthopedics referral was submitted for additional evaluation. His vital signs in the OHU on the day of the incident were recorded as follows: blood pressure 132/88 mmHg, temperature 36.2°C, pulse 86 beats per minute, respiratory rate 20 breaths per minute, and oxygen saturation 100% on room air. 

Within one week of the initial injury, the patient returned to the OHU for follow-up care. At that visit, his blood pressure had decreased to 130/91 mmHg, his temperature was 36°C, his pulse had risen slightly to 89 beats per minute, and his respiratory rate remained steady at 20 breaths per minute. His oxygen saturation continued to be 98% on room air. A random blood sugar (RBS) test performed during this follow-up recorded a value of 90 mg/dL. The patient reported that his left foot continued to be swollen and painful. His foot was redressed and bandaged during this appointment, and no cast was deemed necessary given the absence of fractures on prior imaging. The wound appeared clean with well-approximated edges, minimal serous drainage, and no signs of infection. The clinical team advised that he adhere to rest, ice, compression, and elevation (RICE) therapy to allow for further healing and reduction of swelling. For pain control, the patient was also prescribed diclofenac 1% gel, based on his preference, to be applied topically twice daily for seven days.

Approximately two weeks after the injury, the patient was re-evaluated at the OHU. At this time, his vital signs measured 130/85 mmHg for blood pressure, 36.1°C for temperature, 88 beats per minute for pulse, with a respiratory rate of 20 breaths per minute and oxygen saturation of 99% on room air. The patient complained that his left foot was still tender to touch and that complete healing had not been achieved. He expressed concern regarding his ability to resume his physically demanding work as a plumber, which requires frequent contact with water, climbing, and heavy lifting. An orthopedics referral was submitted for additional evaluation.

Approximately three weeks into the recovery period, the patient was seen again at the OHU. His vital signs on this visit were a blood pressure of 127/89 mmHg, pulse of 77 beats per minute, temperature of 36.3°C, and respiratory rate of 20 breaths per minute, with oxygen saturation remaining at 100% on room air. By this stage, the laceration had healed further and sutures were previously removed at a primary care provider. However, the patient continued to experience pain and there was mild tenderness on examination over the third toe during flexion and extension movements. The earlier numbness in the first two toes had resolved by this point. The patient was advised to continue with the prescribed analgesics, paracetamol and ibuprofen, and to remain on the RICE regimen. He was also instructed to perform gentle range-of-motion and strengthening exercises to promote further recovery. Follow-up appointments were scheduled to ensure timely monitoring of his progress and to make any necessary adjustments to his treatment plan.

**Figure 2:**
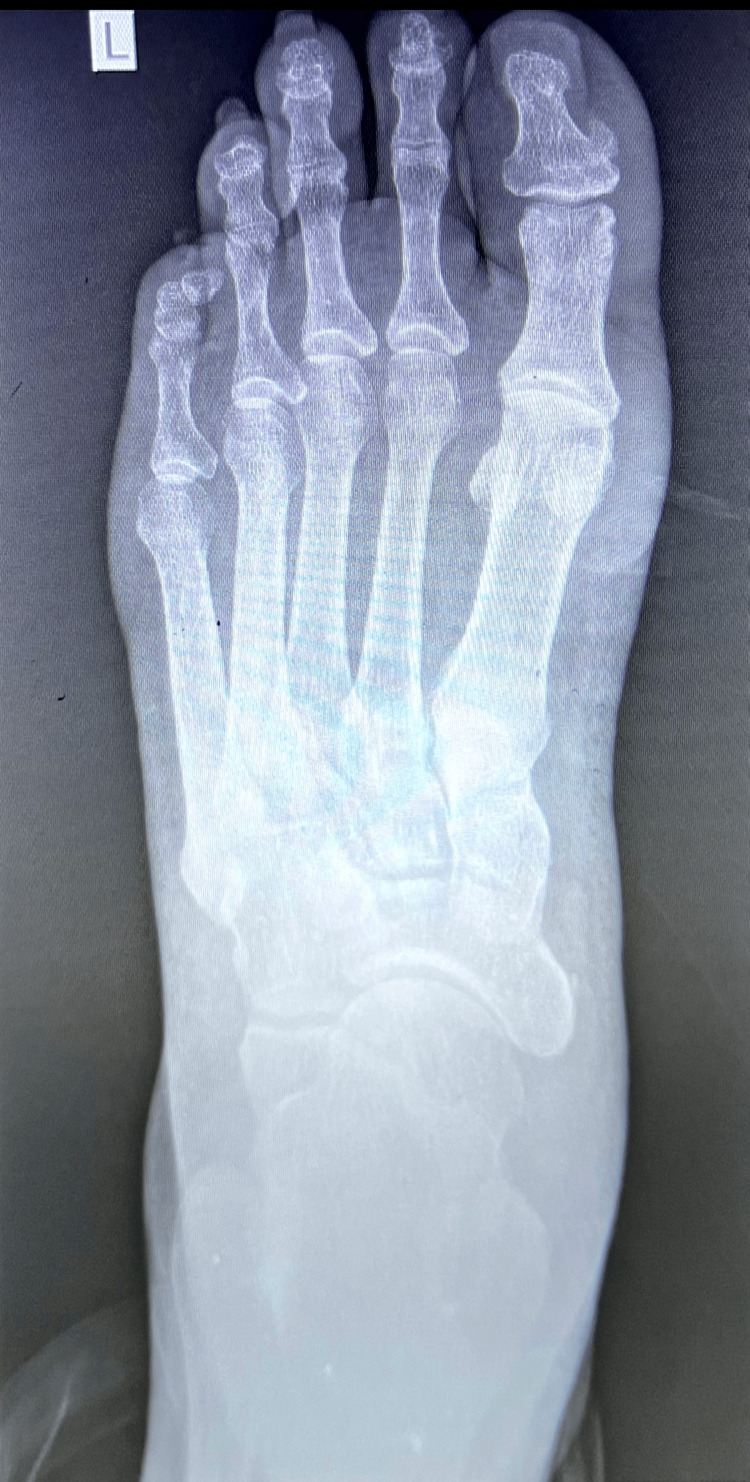
Follow‑up AP plain X-ray of the left foot at four weeks post‑injury showing no fractures. AP: anteroposterior.

**Figure 3:**
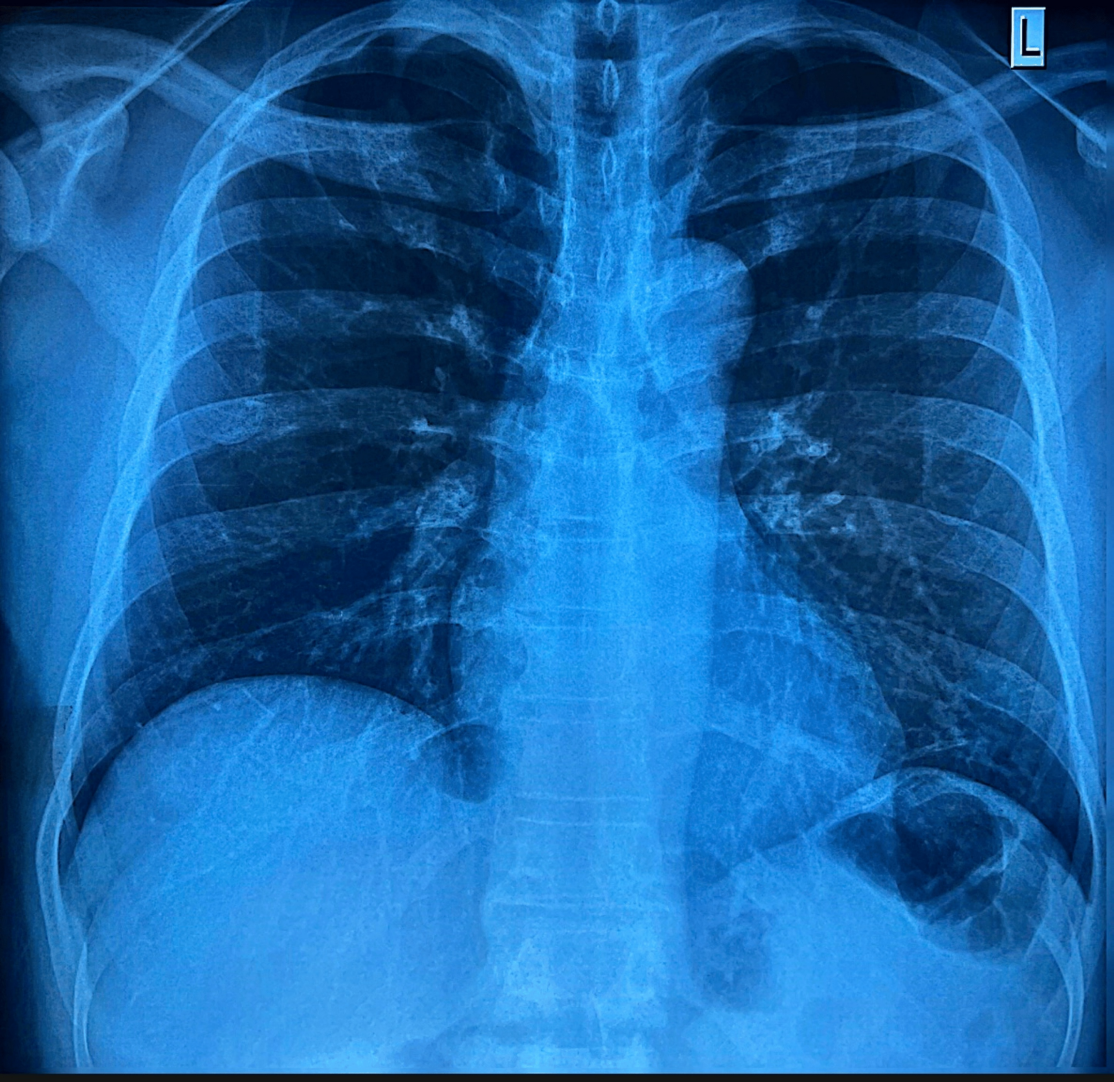
PA plain X-ray of the chest at four weeks post‑injury with no acute thoracic abnormalities. PA: posteroanterior.

**Figure 4:**
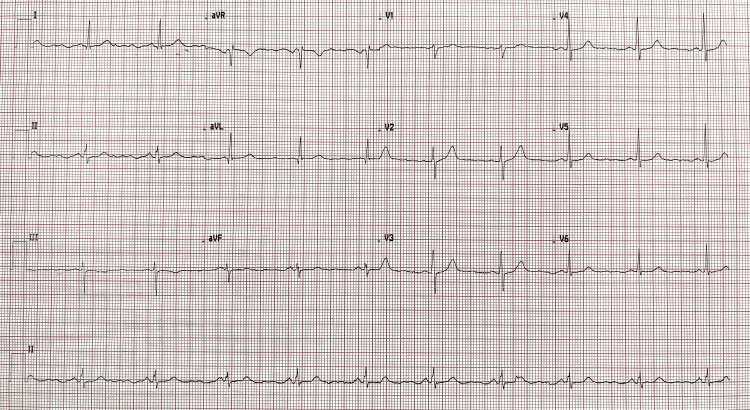
Electrocardiogram at four weeks post‑injury showing normal sinus rhythm at 60 beats per minute in all leads.

By the end of the fourth week from the time of the injury, the patient returned for another detailed assessment at the OHU. At this follow-up, a comprehensive wellness evaluation was performed, including a plain X-ray of the chest, urine dipstick testing, and electrocardiogram (ECG). His temperature was recorded at 36.2°C, blood pressure was 128/89 mmHg, and his pulse measured 84 beats per minute. The respiratory rate was 20 breaths per minute, and his oxygen saturation improved to 100% on room air. A repeat RBS test showed a value of 96 mg/dL. Urine dipstick analysis yielded no abnormal findings, while his body mass index (BMI) was calculated at 23.0, which is considered normal. During this assessment, the patient described persistent pain in the first three toes of his left foot. He described the pain as shooting in nature, rating its severity as 6 out of 10. The pain was intermittent and was aggravated by prolonged standing and bending of the toes. Due to delayed healing and lack of facilities to measure serum B-vitamin levels, empirical vitamin B supplements were initiated at week 4 for one month to support nerve recovery. He found temporary relief when he used Flamar MX (combination of Diclofenac, Paracetamol, and Chlorzoxazone) tablets on an as-needed basis. Additionally, he reported experiencing numbness in the first two toes, localized to the dorsal aspect. However, this numbness had gradually improved with rest and elevation of the foot. In our resource-limited setting and given his persistent symptoms, a repeat plain X-ray of the left foot was requested to reassess healing progress. The radiograph, shown in Figure 2, ruled out fractures. At the same time, plain X-ray of the chest revealed no abnormalities (Figure 3). Electrocardiogram (ECG) revealed a sinus rhythm with a heart rate of 60 beats per minute and no acute changes (Figure 4).

During this fourth-week follow-up, a comprehensive laboratory work-up was conducted. The investigations included a complete blood count (CBC), basic metabolic panel, lipid profile, liver function tests, and endocrine evaluations. The CBC results, which are summarized in Table 1, were within normal limits, ruling out the presence of an active infection or significant inflammatory response. The basic metabolic panel and electrolyte measurements, detailed in Table 2, were also normal. The lipid profile, as presented in Table 3, showed elevated total cholesterol and low-density lipoprotein (LDL) levels. Although these findings were not directly related to the acute injury, they were noted for long-term health management. Liver function tests and additional biochemical parameters, as summarized in Table 4, were unremarkable. Endocrine evaluations, including thyroid-stimulating hormone (TSH), free thyroxine (FT4), and prostate-specific antigen (PSA) levels, as well as fasting glucose and hemoglobin A1c (HbA1c) values, are detailed in Table 5. Additional findings such as the results of the urine dipstick exam and body mass index (BMI) are summarized in Table 6. These laboratory investigations confirmed that no systemic conditions were impeding the wound healing process. The clinical team maintained the current management plan and decided to resume the patient's duties with restrictions. The patient was restricted from climbing or standing for more than one hour. A subsequent review was arranged for approximately one week later. In light of the patient's elevated LDL (156.94 mg/dL), rosuvastatin 20 mg once nightly was started. The patient was referred to a dietician for nutritional counselling. The lipid profile will be rechecked in six months to guide dose reduction or therapy withdrawal.

**Table 1:** Complete blood count at four weeks post‑injury. WBC: white blood cell; UWBC: universal white blood cell; RBC: red blood cell; Hb: hemoglobin; HCT: hematocrit; MCV: mean corpuscular volume; MCH: mean corpuscular hemoglobin; MCHC: mean corpuscular hemoglobin concentration; RDW: red blood cell distribution width; RDW-SD: red blood cell distribution width standard deviation; PLT: platelets; MPV: mean platelet volume.

Test	Result	Reference range
WBC (10³/μL)	5.90	4.00–10.00
UWBC (10⁹/L)	5.90	–
RBC (10⁶/μL)	4.94	4.50–5.50
Hb (g/L)	153.0	130.0–170.0
HCT (%)	42.50	40.00–50.00
MCV (fL)	85.90	83.00–101.00
MCH (pg)	30.90	27.00–32.00
MCHC (g/L)	360.00	–
RDW (%)	13.90	11.60–14.00
RDW-SD (fL)	42.40	–
PLT (10⁹/μL)	319	150–410
MPV (fL)	8.00	6.90–10.80
Neutrophil (%)	41.70	40.00–60.00
Lymphocyte (%)	48.10	20.00–40.00
Monocyte (%)	7.70	2.00–10.00
Eosinophil (%)	1.70	1.00–6.00
Basophil (%)	0.80	1.00–2.00
Absolute neutrophils (10³/μL)	2.50	2.00–7.00
Absolute lymphocytes (10³/μL)	2.90	1.00–3.00
Absolute monocytes (10³/μL)	0.50	0.20–1.00
Absolute eosinophils (10³/μL)	0.10	0.02–0.50
Absolute basophils (10³/μL)	0.00	0.00–0.10

**Table 2:** Basic metabolic panel and electrolytes at four weeks post‑injury. BUN: blood urea nitrogen.

Test	Result	Reference range
Sodium (mmol/L)	139.0	136.0–145.0
Potassium (mmol/L)	4.3	3.5–5.1
Chloride (mmol/L)	102.0	98.0–107.0
BUN (mg/dL)	11.6	6.0–23.0
Creatinine (mg/dL)	1.0	0.7–1.2
Uric acid (mg/dL)	2.90	2.40–5.70
Magnesium (mg/dL)	2.1	1.6–2.6
Calcium (mg/dL)	9.7	8.6–10.2
Phosphorus (mg/dL)	3.73	2.50–4.50

**Table 3:** Lipid profile at four weeks post‑injury. HDL: high-density lipoprotein; LDL: low-density lipoprotein

Test	Result	Reference range (mg/dL)	
Cholesterol	247.00	50.00–200.00	
HDL	57.70	35.00–55.00	
Triglycerides	161.80	0.00–150.00	
LDL	156.94	0.00–100.00	

**Table 4:** Liver function and biochemistry panel at four weeks post‑injury. AST: aspartate transaminase; ALT: alanine aminotransferase; GGT: gamma-glutamyl transpeptidase.

Test	Result	Reference range
AST (U/L)	23.0	5.0–40.0
ALT (U/L)	30.0	5.0–41.0
GGT (U/L)	35.00	10.00–71.00
Alkaline phosphatase (U/L)	88	40–129
Total protein (g/dL)	7.9	6.6–8.7
Albumin (g/dL)	4.63	3.50–5.20
Globulin (g/dL)	3.2	2.3–4.5
Total bilirubin (mg/dL)	0.46	0.12–1.40
Direct bilirubin (mg/dL)	0.14	0.00–0.40
Indirect bilirubin (mg/dL)	0.32	0.10–1.00

**Table 5:** Urinalysis and BMI at four weeks post‑injury. BMI: body mass index.

Test	Result	Comment
Urinalysis	Normal	No abnormalities detected
Body Mass Index (BMI)	23.0	Within normal range

**Table 6:** Endocrine and metabolic tests at four weeks post‑injury. TSH: thyroid-stimulating hormone; FT4: free thyroxine; PSA: prostate-specific antigen; HbA1c: hemoglobin A1C.

Test	Result	Reference range
TSH (UIu/mL)	0.56	0.27–4.20
FT4 (ng/dL)	1.07	0.93–1.70
Total PSA (ng/mL)	0.480	0.100–4.000
Fasting glucose (mg/dL)	92	74–109
HbA1c (%)	5.6	4.8–5.9

By roughly five weeks from the time of the injury, the patient reported further improvements in his condition. Orthopedics evaluated him a day prior to this OHU visit and confirmed no fractures, discharging him from follow-up. Radiology reports for both his initial and four‑week foot radiographs (Figures 1, 2) confirmed the absence of bony injury. His vital signs were stable, with a temperature of 36.1°C, a pulse of 78 beats per minute, a respiratory rate of 20 breaths per minute, and a blood pressure of 124/82 mmHg. Oxygen saturation was maintained at 99% on room air. At this point, the patient confirmed that the laceration had completely healed and that he was able to ambulate for more than three hours without significant discomfort. He continued to follow a rehabilitation program under the guidance of the PMR team. This involved daily strengthening and range-of-motion exercises, including seated heel raises, towel toe curls, ankle dorsiflexion and plantar flexion drills, as well as calf stretches. These exercises were performed once daily, with three sets of ten repetitions each, progressing in intensity as tolerated. His analgesia regimen was adjusted to include tramacet (combination of tramadol and paracetamol) for breakthrough pain, and vitamin B supplements were continued to support nerve recovery. The patient was cleared to resume full work duties without restrictions, with plans for further monitoring in a follow-up appointment approximately two weeks later.

At the final visit, around seven weeks after the injury, the patient returned for a comprehensive review at the OHU. His physical examination was unremarkable. He was discharged from PMR follow-up because his symptoms had resolved adequately to permit a return to his normal work duties. The patient no longer experienced pain with prolonged standing or walking, and his gait was normal. The patient was advised to continue routine exercises and to seek follow-up care if any new or recurring symptoms developed.

Throughout this clinical journey, the patient’s progress was continuously evaluated using serial imaging studies and detailed clinical assessments. In our resource-limited setting, we relied on serial imaging. The initial plain X-ray at the AED (Figure 1) ruled out any fracture. A follow-up plain X-ray at four weeks (Figure 2) confirmed that the foot was fracture-free. The plain X-ray of the chest (Figure 3) and the ECG (Figure 4) provided additional reassurance regarding his comprehensive wellness assessment. Moreover, the laboratory data summarized in Tables 1 through 6 supported the clinical findings and confirmed that no systemic conditions were affecting his recovery.

The management of this case followed a structured, time-framed approach that began with immediate wound care, including the use of 1 liter of normal saline for irrigation, placement of eight 5-0 nylon sutures for laceration repair, administration of tetanus prophylaxis, and prescription of prophylactic antibiotic therapy with amoxicillin-clavulanate along with pain control using ibuprofen. Subsequent evaluations helped monitor the healing process, with serial imaging studies and laboratory tests guiding further management. This coordinated approach, involving the AED, OHU, orthopedics, radiology, dietetics, and the PMR team, ensured that the patient received comprehensive care and was able to gradually return to full occupational duties with minimal long-term complications.

## Discussion

Management of this patient hinged on several evidence‑based steps. Irrigation with a high volume of fluid is the most important measure to reduce infection risk. Literature reports irrigation volumes of 50-100 mL per centimeter of laceration length [11]. For a 10 cm wound (requiring 500-1000 mL), we used 1 L of normal saline. Studies comparing tap water to sterile saline found no significant difference in infection rates for simple wounds, but in cases with heavy contamination, sterile saline remains the standard [12]. Pressurized irrigation systems, such as syringe or canister devices, deliver fluid under controlled pressure and may further enhance wound cleansing without damaging tissue [13]. 

Selection of suture material and technique influences healing and complication rates. We chose simple interrupted 5‑0 nylon sutures and placed eight stitches to achieve precise edge alignment. Nylon is nonabsorbable and induces minimal tissue reaction, yielding better cosmetic results and lower risk of suture‑related inflammation [13]. Interrupted sutures also permit local drainage should infection arise, whereas continuous techniques risk forming a closed space that can harbor bacteria [14].

RICE therapy remains foundational for acute soft‑tissue injury. Although high‑quality trials are scarce, systematic reviews suggest that cryotherapy combined with compression and elevation reduces pain and swelling more than rest alone [15]. Intermittent icing, applying ice for 10-15 minutes followed by rest, may offer better comfort than continuous cold exposure [16]. Compression bandages and elevation further limit edema, supporting tissue perfusion and nutrient delivery.

Effective analgesia is critical to support wound care and early mobilization. We provided ibuprofen 400 mg every eight hours as needed. NSAIDs like ibuprofen relieve pain and reduce inflammation with a favorable safety profile for short‑term use [17]. When combined with topical NSAIDs, such as diclofenac 1% gel applied topically twice daily, analgesic efficacy may improve without raising systemic adverse effects [18].

Tetanus prophylaxis was administered with both toxoid vaccine and human tetanus immune globulin. Current recommendations advise tetanus immunoglobulin for any wound in a patient who has not received a booster in the previous five years, along with a toxoid booster to ensure active immunity [14]. Prompt administration reduces the risk of tetanus, which remains a concern in deep or contaminated wounds.

Antibiotic prophylaxis is debated in laceration management, but guidelines support its use in heavily contaminated wounds or when tetanus status is uncertain. We prescribed amoxicillin‑clavulanate 875/125 mg twice daily for seven days. This regimen covers common skin flora and anaerobes found in foot injuries [19]. Meta‑analyses of surgical prophylaxis demonstrate that amoxicillin‑clavulanate reduces postoperative infection risk more effectively than narrow‑spectrum agents in mixed aerobic‑anaerobic wounds [19].

Imaging follow‑up was essential given the patient’s persistent symptoms. Initial and four‑week foot radiographs (Figures 1 and 2) ruled out fractures. Repeat imaging was utilized due to the resource-limited setting and persistent pain beyond the expected healing timeframe [14]. A routine chest radiograph (Figure 3) and an ECG (Figure 4) were performed as part of a comprehensive wellness assessment.

Occupational considerations shaped our management plan. Foot injuries in manual laborers can lead to prolonged work absence and financial hardship. This rationale supported the patient’s extended sick leave to ensure complete healing. Early referral to occupational health, appropriate sick-leave duration, and gradual return to light duties help prevent reinjury and support economic stability. Coordinated communication with the patient’s employer ensured appropriate job modifications, such as avoiding heavy lifting and prolonged standing, while protecting the healing tissue.

Physical rehabilitation under the guidance of a PMR specialist played a central role. Controlled strengthening and range-of-motion exercises began during the early post‑injury period, aligning with evidence that early mobility improves functional outcomes compared to strict immobilization [16]. By providing detailed rehabilitation prescriptions, this case fills a gap in existing literature by specifying physical therapy parameters and reinforces guideline recommendations for structured early mobilization [16]. These helped restore ankle flexibility and foot muscle support. 

Comprehensive laboratory evaluation ruled out systemic factors that could impede healing. The complete blood count, metabolic panel, liver function tests, and endocrine studies (Tables 1-6) were all within normal limits, excluding anemia, renal insufficiency, hepatic dysfunction, diabetes, or thyroid disease. Identifying and managing lipid abnormalities as part of general health maintenance did not alter the acute treatment but underscored the value of holistic care in occupational health settings.

Multidisciplinary collaboration proved vital. Emergency physicians performed initial wound care and imaging. Radiology provided expert interpretation of each study, ensuring accurate fracture exclusion. Occupational health specialists oversaw follow-up evaluations, job-site risk assessments, and sick-leave coordination. Orthopedics confirmed the absence of fractures. The dietician offered nutritional counselling and crafted a plan to address his lipid abnormalities. The PMR team directed rehabilitation protocols. This integrated approach facilitated timely adjustments to the management plan, such as modifying analgesic regimens and advancing rehabilitation exercises.

The stepwise, time‑framed strategy supported favorable healing. Immediate irrigation, precise suturing, prophylactic antibiotics, and NSAID‑based analgesia created optimal conditions for tissue repair. Serial follow‑up of imaging and detailed clinical assessments guided adjustments in rest periods and rehabilitation intensity. Gradual progression from duties with restrictions to full work responsibilities minimized the risk of setbacks while promoting functional recovery.

Superficial soft-tissue lacerations typically heal in four to six weeks [20]. In our patient, complete recovery occurred between his week-5 and week-7 OHU visits, which aligns with this timeframe. Prompt presentation to the OHU and AED within two hours allowed for early irrigation and timely suturing, optimizing the healing environment and supporting this result.

This case illustrates how combining established clinical practices and targeted interventions can achieve efficient healing in occupational foot injuries. Rigorous wound cleansing, appropriate suture selection, antibiotic coverage in high‑risk wounds, RICE with early exercise, and structured rehabilitation underpin successful outcomes. Regular assessment, both clinical and diagnostic, ensures that interventions remain aligned with the patient’s progress and work demands. By applying these measures, healthcare teams can help patients return to their occupations safely and with minimal long‑term disability.

## Conclusions

Effective management of occupational foot injuries combines thorough wound care, timely imaging, and targeted rehabilitation. High‑volume saline irrigation and precise suturing set the stage for healing. Antibiotic prophylaxis and tetanus immunization reduce infection risk. Pain control with NSAIDs and structured RICE therapy support early mobilization. Serial assessments (clinical and radiographic) guide adjustments in treatment intensity. Coordinated care across emergency, occupational health, orthopedics, and rehabilitation services enables safe return to work. This stepwise approach minimizes complications, restores function, and helps manual laborers resume their duties with confidence.
